# SARS-CoV-2 Vaccines: Inactivation by Gamma Irradiation for T and B Cell Immunity

**DOI:** 10.3390/pathogens9110928

**Published:** 2020-11-10

**Authors:** Arno Mullbacher, Julian Pardo, Yoichi Furuya

**Affiliations:** 1 Department of Immunology and Infectious Disease, John Curtin School for Medical Research, Australian National University, Canberra 0200, ACT, Australia; arno.mullbacher@anu.edu.au; 2 Immunotherapy, Inflammation and Cancer, Biomedical Research Centre of Aragon, ARAID/Aragon Health Research Institute (IIS Aragon)/University of Zaragoza, 50009 Zaragoza, Spain; pardojim@unizar.es; 3 Department of Immunology and Microbial Disease, Albany Medical College, Albany, NY 12208, USA

**Keywords:** SARS-CoV-2, COVID-19, Gamma-ray irradiated vaccine

## Abstract

Despite accumulating preclinical data demonstrating a crucial role of cytotoxic T cell immunity during viral infections, ongoing efforts on developing COVID-19 vaccines are mostly focused on antibodies. In this commentary article, we discuss potential benefits of cytotoxic T cells in providing long-term protection against COVID-19. Further, we propose that gamma-ray irradiation, which is a previously tested inactivation method, may be utilized to prepare an experimental COVID-19 vaccine that can provide balanced immunity involving both B and T cells.

In excess of 100 experimental vaccines against COVID-19 are in development, of which more than 40 are currently being evaluated in clinical trials [1]. Primarily, these experimental vaccines are focused on induction of virus-neutralizing antibodies using various vaccine technology platforms such as synthetic spike proteins and viral vectors. This is not surprising, given that historically antibodies have been used as an immune correlate of vaccine-mediated protection against infectious diseases. However, given our understanding of SARS-CoV-2, one would predict that CD4^+^ T cells and CD8^+^ cytotoxic T (Tc) cells will play an important role in facilitating recovery and protection against severe COVID-19. The potential benefit of additional Tc cell-mediated immunity is not only in preventing deaths but also in minimizing the risk of transmission to others by reducing viral load and thus shedding during the recovery phase.

The important feature of an effective vaccine is induction of long-lived immunity. A recently published non-human primate study demonstrated that primary SARS-CoV-2 infection provides protection against homologous re-infection 4 weeks after the initial exposure, and this protection was found to be associated with neutralizing antibodies [2]. However, while achieving sterilizing immunity via induction of neutralizing antibodies appears feasible, growing concerns exist that protective levels of neutralizing antibodies do not persist. Numerous clinical studies have shown that antigen-specific antibodies and neutralizing antibodies against SARS, a disease caused by a closely related coronavirus, can wane rapidly within 1-3 years among survivors of the 2003 SARS epidemic [3,4]. It is noteworthy that human coronaviruses, although less virulent, have been causing seasonal infections, much like influenza, which indicates that either viral mutation enables coronaviruses to evade antibodies and/or antibody-mediated protection is short-lived [5]. Indeed, studies show that reinfection with seasonal coronaviruses occurs frequently, with protective immunity lasting as little as 80 days [6,7]. Similarly, an early study on SARS-CoV-2 reported that some individuals who apparently recovered from laboratory-confirmed COVID-19 have tested positive a second time, an indication of potential reinfection [8,9]. Furthermore, a recent serological study reported that 40% and 12.9% of asymptomatic and symptomatic individuals, respectively, became seronegative within 2–3 months after infection [10]. Therefore, unless the vaccine induces immunity more efficiently than the live virus, one would expect that vaccine-induced antibodies will likewise be short lasting. Achieving sterilizing immunity by antibody-based vaccines may prove difficult. 

Given the above, Tc cell immunity against coronaviruses may present an important aspect of any successful vaccine as it is thought to be long-lived. Recent studies have detected SARS-CoV-2 reactive T cells that are capable of expressing IFNγ and/or granzyme B in 40~60% of uninfected individuals [11,12]. Notably, this percentage increased to 80–100% for convalescent COVID-19 patients, suggesting that (1) unlike antibodies, Tc cells against coronaviruses persist in humans and can be detected months and/or years after an infection; (2) memory Tc cells are directed against conserved regions of coronaviruses and therefore are broadly cross-reactive; and (3) specific T cell responses are associated with recovery from SARS-CoV-2 infection. An implication based on these observations is that coronaviruses will be less likely to evade Tc cell immunity due to MHC class I polymorphism. Indeed, a potential benefit of T cell responses was recently demonstrated in two clinical studies that found an inverse correlation between severe COVID-19 and lower CD4^+^ and CD8^+^ T cell blood counts on admission [13,14]. Thus, there are realistic benefits for considering T cell-based vaccination approaches against COVID-19. Future studies are needed to determine whether Tc cell immunity influences the clinical course of SARS-CoV-2 infection and whether the presence of SARS-CoV-2 reactive Tc cells prior to an infection correlates with the development of less severe COVID-19. It is important to note, however, that T-cell-based vaccines will unlikely provide sterilizing immunity against SARS-CoV-2 since cytotoxic T cells will engage after an infection of host cells. Therefore, an ideal COVID-19 vaccine should stimulate both B and T cell immunity to complement each other to provide optimal protection against severe COVID-19. 

Several forms (inactivated, recombinant protein, DNA, RNA, and viral-vector-based) of COVID-19 vaccine candidates are currently being evaluated in clinical trials. Whether these experimental vaccines are capable of providing protective T cell immunity in humans is currently unknown and should be investigated. In particular, DNA- and RNA-based vaccines, in theory, should generate MCH-I antigens in situ in a manner similar to natural viral infection. However, it should be noted that DNA vaccines have been tested against other infectious diseases in humans and have failed in the past to demonstrate the induction of strong immune responses [15,16,17]. As for inactivated and purified viral vaccines, a substantial amount of data exists in the literature that inactivated antigens are mainly dependent on the MHC-II pathway and therefore they are not anticipated to mount strong MHC-I-restricted immune responses [18,19,20]. Lastly, although several viral vectors are currently approved for human use, several challenges exist for their application. These include (1) immunodominance of the vector genes over transgenes; and (2) pre-existing anti-vector immunity due to natural exposure to the virus or induction of anti-vector immunity upon first use, which would not permit effective use of the same viral vector in the same patient. 

Generally, the generation of Tc cell immunity requires live viral infection, and therefore most viral inactivation methods eliminate T cell immunogenicity. However, early studies in the 1970s and 80s have identified gamma-irradiation as a superior inactivation method that can better preserve T cell immunogenicity relative to other inactivation procedures. This favorable characteristic of gamma-irradiation can be attributed to the high penetrative strength of gamma-rays that cause direct damage to genetic material without altering structural proteins [21]. Therefore, gamma-irradiated viruses should be able to infect host cells without producing infectious progeny. Indeed, we have previously demonstrated that alphaviruses [22] and bunyaviruses [23] can be rendered non-infectious by gamma-irradiation and yet still possess the capacity to generate cytotoxic T cell responses. We later applied gamma-irradiation to prepare an experimental influenza vaccine and reported that gamma-ray irradiated sterile influenza virus preparations promote Tc cell immunity [24,25,26,27]. Most importantly, gamma-ray irradiated influenza virus preparations were highly protective against heterologous influenza infections, including H5N1 avian flu, in mice, and this T cell memory was found to be long-lived [24,25,26,28]. This experimental protocol of using low dose-rate sterilizing gamma irradiation of whole viruses for induction of long-lasting Tc cell immunity has been shown to be generally applicable if the immunodominant Tc cell determinants are located in structural virus proteins [24]. This approach may enable the SARS-CoV-2 spike protein to enter the class I MHC antigen presentation pathway [11].

The obvious strength of a gamma-ray irradiation approach is that virus replication can be eliminated while preserving its infectivity and immunogenicity, thus presenting the viral proteins to the immune system in a natural way, facilitating the induction of both T cell and humoral immunity. In addition, vaccine manufacturing has been greatly simplified, solely requiring cell culture virus growth, purification, and low-dose inactivation using a gamma ray source generally readily available for sterilization procedures of commercial products. Lastly, our previous work has also demonstrated an antigen dose sparing effect of gamma-irradiated virus in mice [27] (discussed in Furuya [29]). This, of course, is important during a pandemic when enough vaccine doses are need to be manufactured with limited production capacity in a short time frame. Therefore, a gamma-ray irradiated virus vaccine could fulfill the unmet need for a safe, cost-effective, widely distributable COVID-19 vaccine.

## References

[1] WHO (2020). DRAFT Landscape of COVID-19 Candidate Vaccines. https://www.who.int/publications/m/item/draft-landscape-of-covid-19-candidate-vaccines.

[2] Deng: W., Bao L., Liu J., Xiao C., Liu J., Xue J., Lv Q., Qi F., Gao H., Yu P. (2020). Primary exposure to SARS-CoV-2 protects against reinfection in rhesus macaques. Science.

[3] Cao W.C., Liu W., Zhang P.H., Zhang F., Richardus J.H. (2007). Disappearance of antibodies to SARS-associated coronavirus after recovery. N. Engl. J. Med..

[4] Liu W., Fontanet A., Zhang P.H., Zhan L., Xin Z.T., Baril L., Tang F., Lv H., Cao W.C. (2006). Two-year prospective study of the humoral immune response of patients with severe acute respiratory syndrome. J. Infect. Dis..

[5] Gorse G.J., Donovan M.M., Patel G.B. (2020). Antibodies to coronaviruses are higher in older compared with younger adults and binding antibodies are more sensitive than neutralizing antibodies in identifying coronavirus-associated illnesses. J. Med. Virol..

[6] Kiyuka P.K., Agoti C.N., Munywoki P.K., Njeru R., Bett A., Otieno J.R., Otieno G.P., Kamau E., Clark T.G., van der Hoek L. (2018). Human Coronavirus NL63 Molecular Epidemiology and Evolutionary Patterns in Rural Coastal Kenya. J. Infect. Dis..

[7] Edridge A.W., Kaczorowska J.M., Hoste A.C., Bakker M., Klein M., Jebbink M.F., Matser A., Kinsella C., Rueda P., Prins M. (2020). Coronavirus protective immunity is short-lasting. medRxiv.

[8] Lan L., Xu D., Ye G., Xia C., Wang S., Li Y., Xu H. (2020). Positive RT-PCR Test Results in Patients Recovered From COVID-19. JAMA.

[9] Tillett R.L., Sevinsky J.R., Hartley P.D., Kerwin H., Crawford N., Gorzalski A., Laverdure C., Verma S.C., Rossetto C.C., Jackson D. (2020). Genomic evidence for reinfection with SARS-CoV-2: A case study. Lancet Infect. Dis..

[10] Long Q.X., Tang X.J., Shi Q.L., Li Q., Deng H.J., Yuan J., Hu J.L., Xu W., Zhang Y., Lv F.J. (2020). Clinical and immunological assessment of asymptomatic SARS-CoV-2 infections. Nat. Med..

[11] Grifoni A., Weiskopf D., Ramirez S.I., Mateus J., Dan J.M., Moderbacher C.R., Rawlings S.A., Sutherland A., Premkumar L., Jadi R.S. (2020). Targets of T Cell Responses to SARS-CoV-2 Coronavirus in Humans with COVID-19 Disease and Unexposed Individuals. Cell.

[12] Le Bert N., Tan A.T., Kunasegaran K., Tham C.Y.L., Hafezi M., Chia A., Chng M.H.Y., Lin M., Tan N., Linster M. (2020). SARS-CoV-2-specific T cell immunity in cases of COVID-19 and SARS, and uninfected controls. Nature.

[13] He R., Lu Z., Zhang L., Fan T., Xiong R., Shen X., Feng H., Meng H., Lin W., Jiang W. (2020). The clinical course and its correlated immune status in COVID-19 pneumonia. J. Clin. Virol..

[14] Liu Z., Long W., Tu M., Chen S., Huang Y., Wang S., Zhou W., Chen D., Zhou L., Wang M. (2020). Lymphocyte subset (CD4+, CD8+) counts reflect the severity of infection and predict the clinical outcomes in patients with COVID-19. J. Infect..

[15] MacGregor R.R., Boyer J.D., Ugen K.E., Lacy K.E., Gluckman S.J., Bagarazzi M.L., Chattergoon M.A., Baine Y., Higgins T.J., Ciccarelli R.B. (1998). First human trial of a DNA-based vaccine for treatment of human immunodeficiency virus type 1 infection: Safety and host response. J. Infect. Dis..

[16] Tacket C.O., Roy M.J., Widera G., Swain W.F., Broome S., Edelman R. (1999). Phase 1 safety and immune response studies of a DNA vaccine encoding hepatitis B surface antigen delivered by a gene delivery device. Vaccine.

[17] Le T.P., Coonan K.M., Hedstrom R.C., Charoenvit Y., Sedegah M., Epstein J.E., Kumar S., Wang R., Doolan D.L., Maguire J.D. (2000). Safety, tolerability and humoral immune responses after intramuscular administration of a malaria DNA vaccine to healthy adult volunteers. Vaccine.

[18] Braciale T.J., Yap K.L. (1978). Role of viral infectivity in the induction of influenza virus-specific cytotoxic T cells. J. Exp. Med..

[19] Blanden R.V. (1974). T cell response to viral and bacterial infection. Immunol. Rev..

[20] Bachmann M.F., Kundig T.M., Kalberer C.P., Hengartner H., Zinkernagel R.M. (1993). Formalin inactivation of vesicular stomatitis virus impairs T-cell- but not T-help-independent B-cell responses. J. Virol..

[21] Lowy R.J., Vavrina G.A., LaBarre D.D. (2001). Comparison of gamma and neutron radiation inactivation of influenza A virus. Antivir. Res..

[22] Mullbacher A., Marshall I.D., Blanden R.V. (1979). Cross-reactive cytotoxic T cells to alphavirus infection. Scand. J. Immunol..

[23] Mullbacher A., Marshall I.D., Ferris P. (1986). Classification of Barmah Forest virus as an alphavirus using cytotoxic T cell assays. J. Gen. Virol..

[24] Mullbacher A., Ada G.L., Hla R.T. (1988). Gamma-irradiated influenza A virus can prime for a cross-reactive and cross-protective immune response against influenza A viruses. Immunol. Cell Biol..

[25] Furuya Y., Chan J., Regner M., Lobigs M., Koskinen A., Kok T., Manavis J., Li P., Mullbacher A., Alsharifi M. (2010). Cytotoxic T cells are the predominant players providing cross-protective immunity induced by {gamma}-irradiated influenza A viruses. J. Virol..

[26] Alsharifi M., Furuya Y., Bowden T.R., Lobigs M., Koskinen A., Regner M., Trinidad L., Boyle D.B., Mullbacher A. (2009). Intranasal flu vaccine protective against seasonal and H5N1 avian influenza infections. PLoS ONE.

[27] Furuya Y., Regner M., Lobigs M., Koskinen A., Mullbacher A., Alsharifi M. (2010). Effect of inactivation method on the cross-protective immunity induced by whole ‘killed’ influenza A viruses and commercial vaccine preparations. J. Gen. Virol..

[28] Mullbacher A. (1994). The long-term maintenance of cytotoxic T cell memory does not require persistence of antigen. J. Exp. Med..

[29] Furuya Y. (2012). Return of inactivated whole-virus vaccine for superior efficacy. Immunol. Cell Biol..

